# Sunscreen products impair the early developmental stages of the sea urchin *Paracentrotus lividus*

**DOI:** 10.1038/s41598-017-08013-x

**Published:** 2017-08-10

**Authors:** Cinzia Corinaldesi, Elisabetta Damiani, Francesca Marcellini, Carla Falugi, Luca Tiano, Francesca Brugè, Roberto Danovaro

**Affiliations:** 1 Department of Sciences and Engineering of Materials, Environment and Urbanistics, Polytechnic University of Marche, Via Brecce Bianche, Ancona Italy; 2 Department of Life and Environmental Sciences, Polytechnic University of Marche, Via Brecce Bianche, Ancona Italy; 3 Ecoreach Ltd., Corso Stamira 61, 60121 Ancona, Italy; 4 Department of Clinical, Specialistic and Odontostomatological Sciences, Polytechnic University of Marche, Via Brecce Bianche, Ancona Italy; 5 Stazione Zoologica Anton Dohrn, Naples, Italy

## Abstract

Marine ecosystems are increasingly threatened by the release of personal care products. Among them, sunscreens are causing concern either for the effects on skin protection from UV radiation and for the potential impacts on marine life. Here, we assessed the UVA protective efficacy of three sunscreens on human dermal fibroblasts, including two common products in Europe and USA, and an eco-friendly product. The sunscreens’ effects were also tested on *Paracentrotus lividus*, a marine species possibly threatened by these contaminants. We found that all tested sunscreens had similar efficacy in protecting human fibroblasts from UVA radiation. Conversely, the sunscreens’ effects on embryo-larval development of *P. lividus* were dependent on the product tested. In particular, the USA sunscreen, containing benzophenone-3, homosalate and preservatives, caused the strongest impact on the sea urchin development, whereas the eco-friendly sunscreen determined the weakest effects. These results suggest that although the tested products protected human skin cells from UVA-induced damage, they might severely affect the success of recruitment and survival of the sea urchin. Our findings underline the importance of developing eco-friendly sunscreens for minimising or avoiding the impact on marine life while protecting human skin from UV damage.

## Introduction

Coastal areas are the most threatened marine regions, being subjected to either direct (contamination, habitat destruction, dumping and marine litter) and indirect (e.g., climate changes) anthropogenic impacts^1, 2^. In the last decade, the study of the impact of micro-pollutants (i.e., synthetic organic and inorganic compounds) has increased considerably^3–5^. Among them, the impact of pharmaceutical and personal care products (PPCPs) has received special attention for the significant impacts determined by their ingredients on different aquatic systems^6, 7^. In particular, production and consumption of sunscreen products is increasing in the cosmetic market on a global scale^8, 9^, and this is causing a parallel increasing concern for marine ecosystem’s health, particularly in areas characterised by the rapid expansion of blue tourism^10^.

Sunscreen products typically contain active ingredients to protect human skin from UV radiation, such as organic compounds that absorb UV rays (e.g. cinnamates, camphor derivatives, benzophenones) and/or inorganic compounds (e.g. TiO_2_ and ZnO), which act as chemical or physical filters preventing or limiting UV penetration. Additional ingredients usually present in almost all commercial products include preservatives, adjuvants, moisturisers and antioxidants. Several sunscreen ingredients have been detected at concentrations of several hundreds of micrograms per litre in the marine environment^3, 11, 12^. Due to the lipophilic nature of these cosmetics^13^, and the insolubility of some of their compounds, sunscreen products tend to bioaccumulate in aquatic animals^14^. The most commonly utilized sunscreens and UV filters, such as cinnamates, benzophenones, as well as the almost ubiquitous preservatives (i.e., parabens), have been tested for their potential impact on some unicellular and pluricellular organisms (including bacteria, phytoplankton, corals and crustaceans) causing effects similar to those reported for other xenobiotic compounds^3, 15, 16^. However, toxicological effects are not entirely responsible for determining the impact of synthetic organic UV filters on marine organisms. Additional studies have revealed that sunscreen ingredients promote viral infections in bacteria and symbiotic algae of tropical corals, causing the bleaching of coral reefs^3, 15^. Furthermore, recent studies have shown that inorganic oxide nanoparticles can be detrimental for marine ecosystems, causing oxidative stress in different organisms and affecting their growth and development^17, 18^. Therefore, the identification of eco-compatible sunscreens able to protect human skin from UV radiation, while preserving marine ecosystems, is of high relevance for minimising the impacts of tourism and recreational activities on marine ecosystems.

In the present study, we investigated the protective efficacy of three different sunscreen products, including two popular brands in Europe and USA and a new product patented as eco-friendly, on human fibroblasts exposed to UVA rays (the most abundant component of UV radiation, responsible for both skin photo-carcinogenesis and photoaging)^19^. In addition, we contextually assessed the effects on the embryonic and larval development of *Paracentrotus lividus*, which represents a key species of coastal ecosystems of the Mediterranean Sea and Eastern Atlantic Ocean and is one of the most common model organisms for ecotoxicological studies^20, 21^. Our findings provide new insights on the effects of sunscreens on marine life and stimulate the use of eco-compatible sun care products, safe for humans and the environment.

## Results

### Photo-stability of sunscreens

The three sunscreens used for this study showed different spectral profiles (Figure S1). These cover the UVB region (290–320 nm) to a similar extent as expected since they are characterized by a high SPF (Sun Protection Factor). Conversely, their UVA absorption spectrum is different depending on the type of UVA filters used. Sunscreens A and C exhibited a maximum absorbance at 350 nm, whereas sunscreen B at the same wavelength absorbed less than 50% of the other two products. Both Sunscreens A and B contain butylmethoxydibenzoylmethane, which is a photo-unstable UV filter but its instability is offset by the co-presence of the photostabilizer, octocrylene. Sunscreen B is characterised by a limited coverage in the same region probably due to the lack of broadband UV filters in its formula, and the low concentration of the UVA filter, butylmethoxydibenozylmethane (1.5%).

After UVA exposure at 275 kJ m^−2^, the three sunscreens resulted photo-stable as no changes in spectral absorbance nor profile were detected.

### Effect of sunscreens on human dermal fibroblasts viability and ROS formation

**Figure 1:**
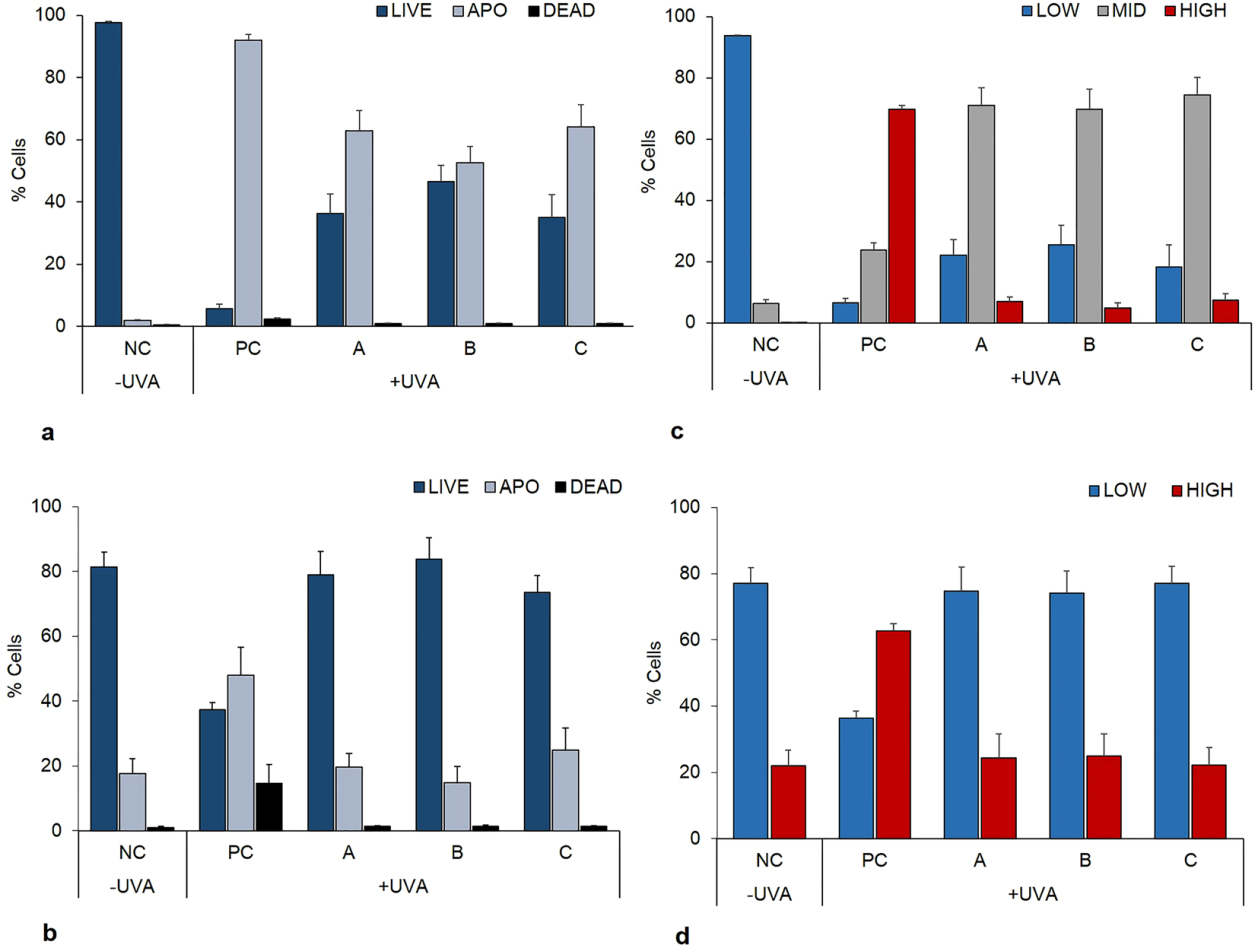
Viability of human dermal fibroblasts (HDF) and their intracellular levels of ROS after exposure to UVA (275 kJ m^−2^). Cell viability, determined by using Guava Via-count dye straight after UVA exposure, time 0 (panel a); cell viability 24 h post-irradiation (panel b); ROS levels determined using carboxy-H_2_DCFDA probe straight after UVA exposure, time 0 (panel c); ROS levels 24 h post-irradiation (panel d). A, B and C indicate the different sunscreens used to screen HDF. A = European sunscreen, SPF 50+ ; B = USA sunscreen, SPF 50; C = Eco-friendly sunscreen, SPF 40. PC = positive control (HDF without sunscreens and exposed to UVA). NC = negative control (unexposed HDF). Error bars represent standard error (n = 5).

The results on the viability of the cells right after exposure to UVA and with the application of the three sunscreens are reported in Fig. 1a. The effect of UVA resulted in an immediate and significant loss of cell viability, associated with a 92% decrease in the fraction of live cells (p < 0.001) and a 90% increase in the number of apoptotic cells (p < 0.001) compared to the negative control. Cell viability, however, partially recovered when the cells were protected by the three sunscreens; in fact, live cells decreased by 50–60% (p < 0.01) with a parallel increase in apoptotic cells (p < 0.01), while the levels of dead ones were no different from the negative control. No significant differences were observed amongst the three sunscreen products. The long-term effect observed on cell viability measured 24 h post-irradiation (Fig. 1b), was a significant decrease in the percentage of live cells (p < 0.001) although lower when compared to the decrease (−44%) observed immediately after irradiation. The limited increase in apoptotic cells ( + 30%) indicated that part of the cells were able to recover from UV damage, whereas 13% died. When protected by the sunscreens, cell’s viability recovered to the levels of the negative control (NC), with no significant differences amongst the three tested products.

The results on ROS formation measured immediately after UVA exposure and 24 h post-irradiation are reported in Fig. 1c,d, respectively. At time 0 (Fig. 1c), there was a significant increase in ROS formation, since the percentage of cells with low levels of ROS decreased by 87% while those with high ROS levels remarkably increased by 70% in the exposed cells (positive control) compared to the unexposed ones (negative control; p < 0.001). However, in the samples protected with the three sunscreens the percentage of cells with high levels of ROS decreased by ten folds, whilst the levels of low and mid ROS (as defined in the Materials and Methods section) increased compared to the positive control (p < 0.05 and p < 0.01, respectively). In particular, the percentage of cells with an intermediate content of ROS (MID) increased three folds in the screened samples (from 18% in the positive control to more than 60% compared to the negative control). No significant differences amongst the three sunscreens were observed. Twenty-four hours post-irradiation, only two distinct regions of fluorescence intensity could be observed (Fig. 1d), hence only two regions were defined for calculating the percentage of cells belonging to each region: Low and High ROS. Despite a slight detoxification, the percentage of cells showing high levels of ROS in the positive control were still significantly higher compared to the negative control (p < 0.01) whereas the cells protected by the sunscreens showed levels of low and high ROS similar to the negative control. No significant differences amongst the three sunscreen products were found.

### Effect of sunscreens on mRNA expression of the extracellular matrix proteins, MMP1 and COL1A1 in human dermal fibroblasts

**Figure 2:**
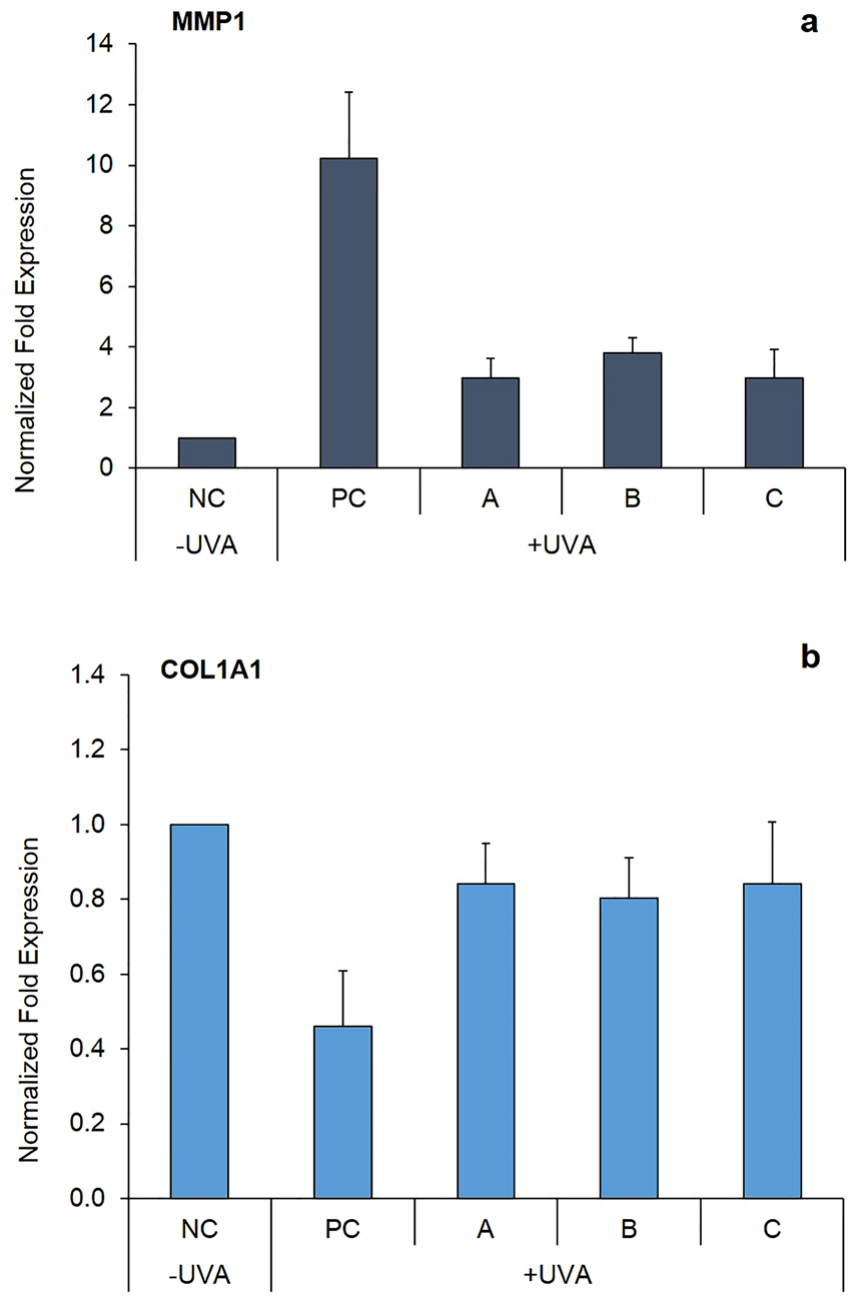
Gene expression analysis of MMP1 (panel a) and COL1A1 (panel b) in human dermal fibroblasts (HDF) exposed to UVA (366 kJ m^−2^), assessed using qPCR 24 h post-irradiation. Data are reported as normalized fold expression using the 2^−∆∆Ct^ method. HDF were screened with sunscreens A (European sunscreen), B (USA sunscreen), C (eco-friendly sunscreen) or with no sunscreen (PC = positive control) and exposed to UVA. NC = negative control, unexposed HDF. Error bars represent standard error (n = 4).

The mRNA expression of the genes MMP1 and COL1A1, measured 24 h post-irradiation, is reported in Fig. 2. The exposure to UVA induced a 10-fold increase in MMP1 expression, which was reduced by the three sunscreens, although it did not reach the levels of the unexposed control (Fig. 2a). No significant differences were observed on comparing the three sunscreens. UVA exposure also determined a 2.5-fold decrease in COL1A1 expression (p < 0.01), which was restored almost completely after application of the three sunscreens. In fact, no significant differences were observed amongst the sunscreens and between sunscreen-treated samples and the negative control (Fig. 2b).

### Impact of sunscreens on the development of zygote and pluteus of *P. lividus*

**Figure 3:**
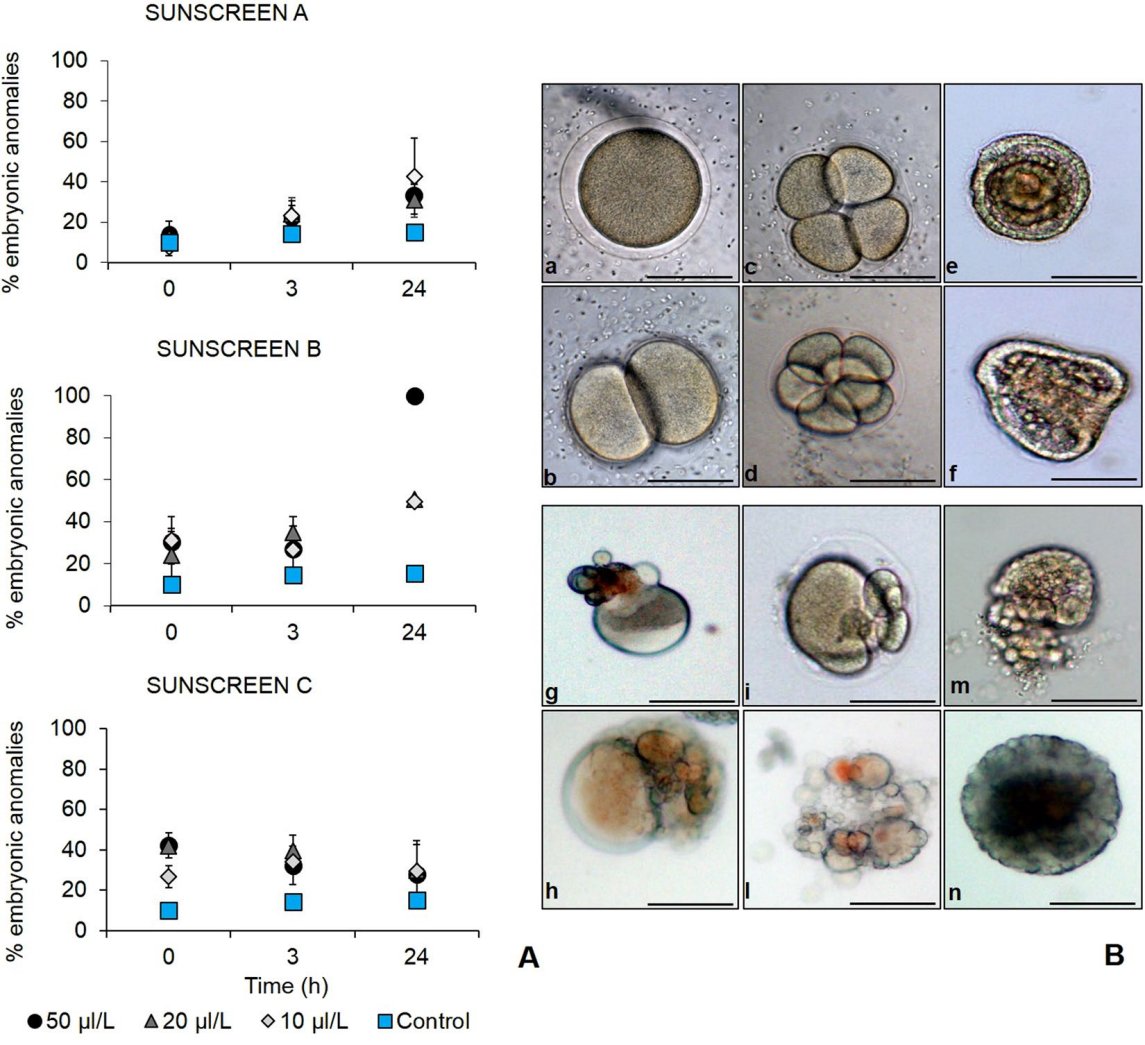
Effect of the sunscreens on the development of embryos of *P. lividus*. **(A)** Percentages of *P. lividus* anomalous embryos after exposure to Sunscreens A, B and C at different concentration (10, 20 and 50 µL L^−1^) over time. Error bars represent standard deviation (n = 3). **(B)** Unexposed embryos at the start of the experiment where the elevated fertilization layer is visible (a); first divisions into blastomeres observed after 70 min (b–d), blastula stage observed after 6 h (e) and gastrula stage observed after 24 h from the beginning of the experiment (f). Abnormal embryos characterized by asymmetrical division into blastomeres and superficial blebs (g–i), signs of cell necrosis (l and n) and esogastrula (m) found in early developmental stages after exposure to sunscreens at the different concentrations. Scale bar: 80 μm.

**Figure 4:**
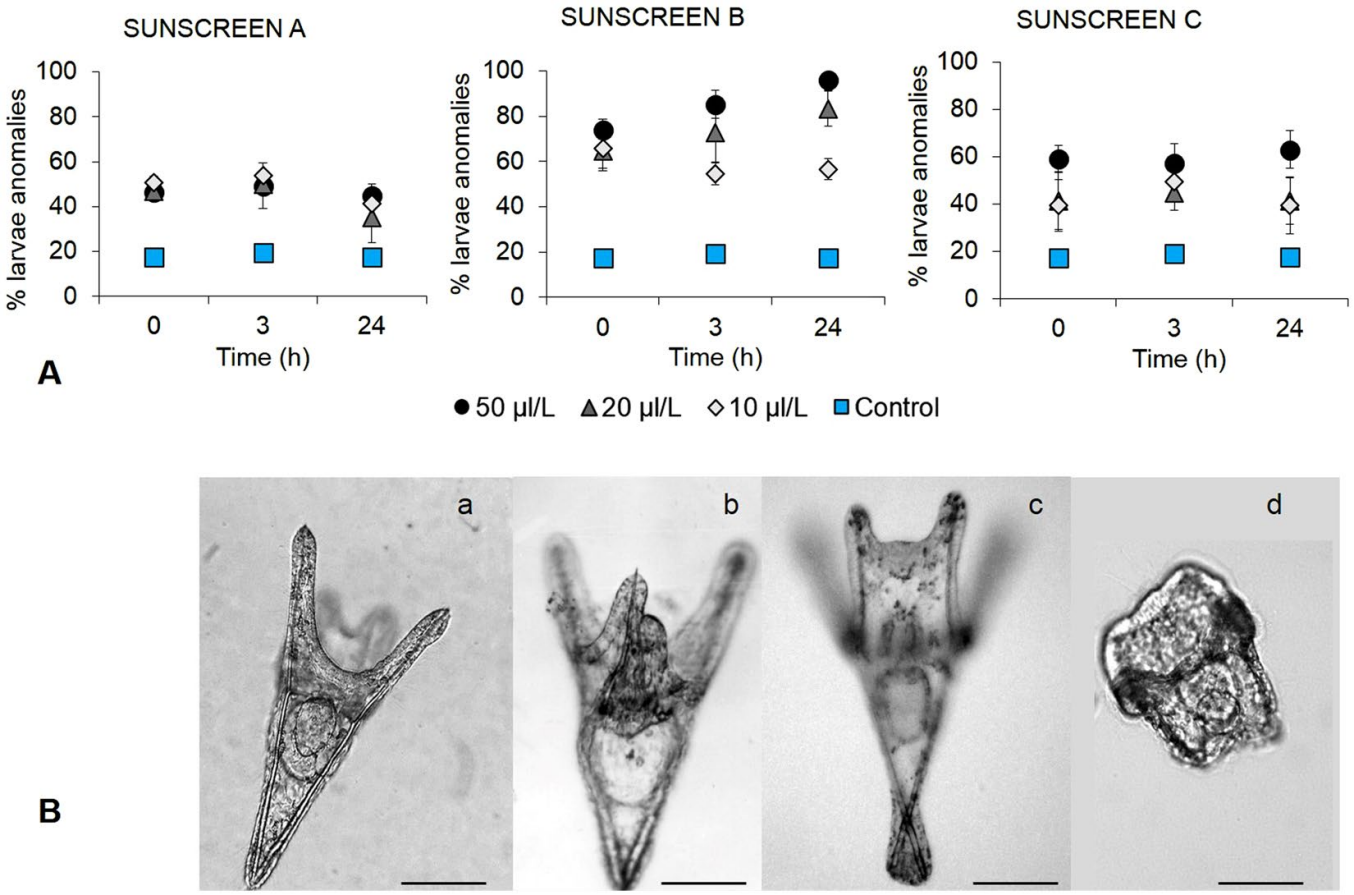
Effect of the sunscreens on the development of larvae of *P. lividus*. (**A**) Percentages of *P. lividus* anomalous larvae after exposure to Sunscreens (**A**,**B** and **C**), at different concentration (10, 20 and 50 µL L^−1^) over time. Error bars represent standard deviation (n = 3). (**B**) Unexposed larva, control (a). Main anomalies found in *P. lividus* larvae after different exposure times: joined anterior arms (b), crossed skeletal tips at the hood apex (c), incomplete skeletal roots (d). Scale bar: 100 μm.

The percentage of anomalous embryos increased significantly over time after addition of Sunscreen A at all of the three concentrations utilised (i.e. 10, 20 and 50 μL L^−1^; p < 0.01, Fig. 3A). The percentage of abnormal embryos increased also after the addition of the different concentrations of Sunscreen B, reaching 100% when they were treated with the highest concentration of sunscreen (50 μL L^−1^; p < 0.001). No significant differences in the percentage of embryonic anomalies were observed among the different concentrations of Sunscreen C and the control at the end of the experiment (after 24 h of exposure). However, significant differences between the different concentrations and the control were observed in the short term (at t_0_, and after 3 h exposure, p < 0.01). The main abnormalities observed in the embryos of *P*. *lividus* in the different treatments included the asymmetrical division into blastomeres, defective gastrulae (esogastrula), non-developed embryos with blebs on the surface and death due to necrosis (ND, Fig. 3B). Therefore, the worst effects of sunscreens resulted in embryos with unrecognizable structures and not compatible with survival. The addition of all sunscreen products, at the different concentrations, determined an immediate increase in the percentage of anomalous larvae already at the beginning of the experiment compared to the control (p < 0.001, Fig. 4A). In the systems treated with Sunscreens A and C the percentage of abnormal larvae remained constant over time at all concentrations tested, whereas in the system added with the highest concentrations of Sunscreen B (20 and 50 µL L^−1^) such a percentage increased after 24 h exposure (p < 0.001), compared to the beginning of the experiment (t_0_). After exposure to the different concentrations of Sunscreen A, we did not find any significant difference in the fraction of anomalous larvae compared to the control. Conversely, after 24 h of treatment (t_24_) at the highest concentrations (20 and 50 µL L^−1^) of Sunscreen B, the fraction of anomalous larvae (ca. 84–97%) was significantly higher than at 10 µL L^−1^ and the control. We also observed that after 24 h of treatment with 50 µL L^−1^ of Sunscreen C, the percentage of anomalous larvae was significantly higher than after treatment with 20 and 10 µL L^−1^ of the same sunscreen (t _24_, p < 0.001). In addition, different types of malformations were distinguished and classified according to the degree of larval alteration to establish the severity of the sunscreen impact, as described in the Materials and Methods section (level 0: normal development; level 1: incorrect location of skeletal rods, level 2: incomplete or absent skeletal rods and level 3: development block at the 4-arms *pluteus*). Based on the percentage of abnormal larvae exposed to Sunscreen A, we found that, at the highest concentration (50 µL L^−1^), ca. 22% of the anomalous larvae fell in the level 3 with a final ISI (i.e., index of sunscreen impact calculated using the frequency of anomalies for each degree of larval alteration at t_24h_) of 1 (slight- moderate impact). On average, at the lowest concentrations (10 and 20 µL L^−1^) of Sunscreen A ca. 20% of the anomalous larvae were classified as levels 1 and 2 resulting in a mean ISI of 0.7 (slight impact, Table 1). After addition of Sunscreen B, we observed that ISI changed depending on the concentration used. Indeed, we found that at the highest concentrations (20 and 50 µL L^−1^), 55–80% of anomalous larvae fell in the level 3, with a final ISI of 2.1–2.7 (high impact), whereas at the lowest concentration (10 µL L^−1^) ISI was 1.4 (moderate impact). Sunscreen C, at the concentration of 50 µL L^−1^, determined a fraction (52%) of anomalous larvae classified as level 1 resulting in an ISI of 0.8 (slight impact). Similarly, at the concentrations of 10 and 20 µL L^−1^, on average ca. 25% of the anomalous larvae were classified as level 1 with a final ISI of 0.6 (slight). The different typologies of larval anomalies encountered after the addition of sunscreens are shown in Fig. 4B. The anomalies observed did not alter the larval body plan but included crossed and/or separated skeletal tips at the hood apex, fused anterior arms, and incomplete or absent skeletal rods.

**Table 1:** Index of Sunscreen Impact (ISI) and environmental impact determined for each sunscreen at the different concentrations based on the level of larval alteration^35, 37, 38^.

Sunscreen A (μL L^−1^)	level^a^	Anomalous larvae (%)	ISI	Environmental impact
50	0	55.2	1.0	Moderate
	1	10.0		
	2	13.0		
	3	21.8		
20	0	65.0	0.7	Slight
	1	10.0		
	2	20.0		
	3	5.0		
10	0	42.3	0.7	Slight
	1	21.3		
	2	15.0		
	3	5.0		
**Sunscreen B** (**µL L** ^−1^)				
50	0	3.5	2.7	High
	1	2.0		
	2	14.5		
	3	80.0		
20	0	16.5	2.1	High
	1	10.0		
	2	18.5		
	3	55.0		
10	0	43.3	1.4	Moderate
	1	6.2		
	2	18.5		
	3	32.0		
**Sunscreen C** (**µL L** ^−1^)				
50	0	36.9	0.8	Slight
	1	52.0		
	2	6.1		
	3	5.0		
20	0	60.2	0.6	Slight
	1	26.0		
	2	8.0		
	3	5.0		
10	0	58.8	0.5	Slight
	1	31.0		
	2	7.2		
	3	3.0		

### AChE activity in *P. lividus*

**Figure 5:**
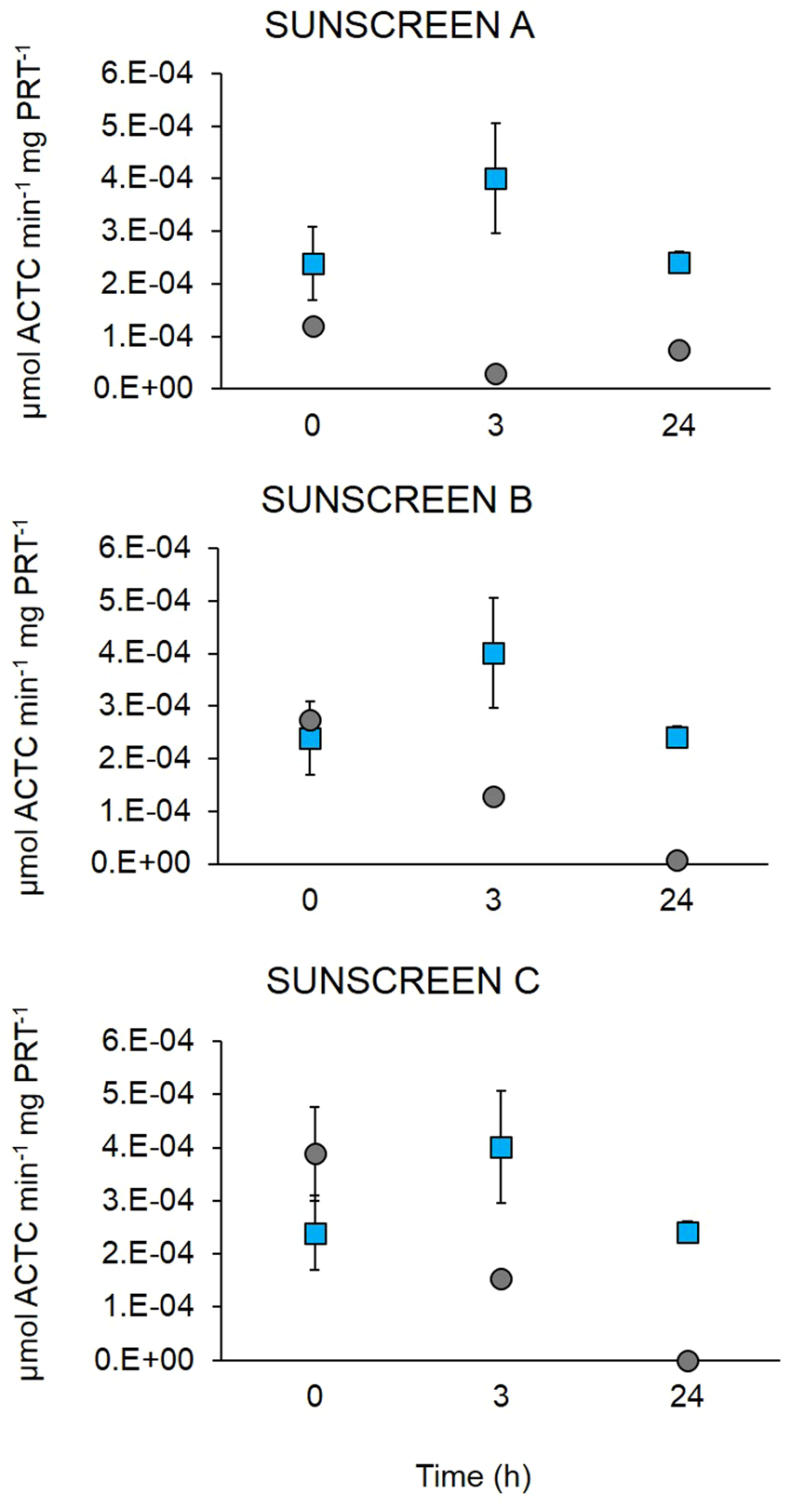
Effects of Sunscreens (**A**,**B** and **C**), at the concentration of 50 µL L^−1^, on AChE activity of larvae of *P. lividus* compared to the control (square symbols). Error bars represent standard deviation (n = 3).

The exposure of the larvae to the different sunscreens (at the highest concentration of 50 µL L^−1^) caused a general decrease over time in AChE activity in all the treatments (Fig. 5). AChE activity in the controls ranged from 2.4 to 4.0 × 10^−4^ μmol mg^−1^ of proteins min^−1^ (at the beginning of the experiment and after 3 h, respectively). Sunscreen A caused an immediate decrease in AChE activity at t_0_ (1.20 × 10^−4^ µmol mg^−1^ of proteins min^−1^, p < 0.01), compared to the control, which further decreased to 0.7 × 10^−4^ µmol mg^−1^ of proteins min^−1^, after 24 h (t_24_, p < 0.001). In the treatment with Sunscreen B, AChE activity decreased significantly after 3 h (t_3_) (1.28 × 10^−4^ µmol mg^−1^ of proteins min^−1^, p < 0.001) and down to 0.07 × 10^−4^ µmol min^−1^mg^−1^ of protein after 24 h (t_24_, p < 0.001) from the beginning of the experiment. Similarly, Sunscreen C decreased significantly after 3 h (1.53 × 10^−4^ µmol mg^−1^ of proteins min^−1^, p < 0.01) and dropped to zero after 24 h of exposure from the beginning of the experiment.

## Discussion

There is an urgent need for developing cosmetic products able to respect marine life and ecosystems while protecting human skin from the risks of solar radiation^22^. A number of studies have investigated the impact of UV filters and preservatives on a variety of marine organisms^3, 12, 23–26^. However, to our knowledge, there are no studies assessing contextually the effects of sunscreens on marine model organisms and human skin cells. Here, we investigated the protective efficacy of two widely used sunscreens in Europe and USA (Sunscreens A and B) containing organic (e.g., benzophenone, homosalate) and inorganic filters (TiO_2_ nanoparticles) already reported to affect marine organisms^13, 16, 17, 27^, and one sunscreen, whose ingredients have been patented as eco-friendly (Sunscreen C)^28^.

Previous investigations have shown that some UV filters contained in commercially available sunscreens are not photo-stable, mainly in the UVA range (320–400 nm), and lose part of their protection when exposed to solar radiation^29, 30^. Among the sunscreens tested here, only Sunscreens A and C, covering both the UVA and UVB regions, can be considered broad-spectrum sunscreens. Although Sunscreen B is characterized by a limited UVA absorption, all of the three sunscreens tested were equally effective in restoring cell viability and reducing ROS production 24 h post-irradiation. Therefore, since antioxidant defenses are able to limit UV-induced ROS production, the application of sunscreens efficiently help HDF in improving their recovery processes. Besides ROS production in cells^31^, UVA radiation is also capable of modulating the gene expression of two genes of interest involved in photoaging, i.e., matrix metalloproteinase-1 (MMP1) and collagen type I, alpha 1 (COL1A1)^32^. The changes in gene expression of COL1A1, which lead to alterations in the synthesis of the alpha-1 chain of type I collagen (the most abundant protein in skin connective tissue), give rise to wrinkle formation, one of the most visible signs of skin photoaging. This protein is down regulated by exposure to UVA, while MMP1, which degrades it, is up-regulated^33^. The experimental tests conducted in this study, reveal that the three sunscreens are effective in reducing the impact of UVA on the extracellular matrix proteins by reducing the amount of UVA rays reaching skin cells. Overall, our results indicate that the three sunscreen products were equally able to protect human dermal fibroblasts from the damage inflicted by UVA radiation. Conversely, the experimental tests performed on the embryonic and larval development of *P. lividus* reveal that the effects of the three sunscreens on embryo and larval development were completely different depending on the product tested. Sunscreen B, at the concentration of 50 µL L^−1^ after 24 h of treatment, caused anomalies in 100% of the embryos of *P. lividus*, which were mostly represented by development block or cell necrosis. The observed impact was dose dependent as, at the lowest concentrations, the development of half of the embryos treated were altered. Sunscreen A had a lower impact than Sunscreen B, affecting the development of one third of the embryos of *P. lividus* at all concentrations. Finally, the effects of Sunscreen C were indistinguishable from the control after 1 day of treatment, suggesting the lack of negative effects on the early development of the investigated sea urchins.

These findings indicate that some of the sunscreens commercially available in Europe and USA contain organic filters and preservatives, which can severely affect the early developmental stages of *P. lividus*, whereas the eco-compatible product does not.

The effects of sunscreens were even more evident on the sea urchin larval development. In particular, the highest concentrations of Sunscreen B, after 24 h of exposure, blocked the development (the most severe anomaly) in most of the larvae observed. Likewise Sunscreens A and C altered the development of the larvae, even if to a lesser extent, indicating that the larval stage of *P. lividus* is more vulnerable than the embryonic one to the impact of sunscreens as also previously reported for other contaminants^34^.

The typologies of anomalies identified in embryos and larvae were similar in all treatments with the different sunscreens, and consistent with those previously reported in larvae of *P. lividus* exposed to inorganic nanoparticles^20, 21, 27^, organophosphate pesticides^35^, and mixtures of contaminants and organic wastes^36^. These results suggest that sunscreens can act as classical pollutants causing alterations in the sea urchin skeleton apparatus, modifying the location of the skeletal rods or determining primary mesenchymal cell migration, and/or potentially inactivating the gene regulatory system, underlying the development of the embryonic skeleton^37^. Based on the available criteria for classifying larval alterations^36, 38, 39^, the effects of Sunscreen B can be referred to an index of impact 3, which represents the most severe toxicity for contaminants, and therefore indicates the presence of a high potential ecological impact. The reason why Sunscreen B determined the worst effect on the embryonic and larval stage of *P. lividus* can be explained by the presence of some exclusive ingredients, such as benzophenone-3 and homosalate and/or preservatives, which have been reported to be highly toxic for marine organisms^40^. In addition, available studies indicate that benzophenone-3 and homosalate, contained in this sunscreen, are potential endocrine disruptors^32, 41^.

In terms of larval development, Sunscreens A and C caused abnormalities in a similar fraction of larvae but with a different degree of morphological alteration. Sunscreen A, at the concentration of 50 µL L^−1^, produced a moderate impact because ca. 35% of the larvae showed the most severe anomalies (i.e. stopping embryo development, causing incomplete or absent skeletal rods, including folded tip and fractured ectoderm), whereas Sunscreen C determined a slight impact on larvae since only 11% of them were severely affected.

Sunscreen A can be detrimental for sea urchins due to the presence of preservatives in its formulation (e.g., benzyl benzoate^42^) and/or TiO_2_ nanoparticles, which have been proven to be a source of hydrogen peroxide in seawater^18^. Sunscreen A also contains the UV filters octocrylene and butylmethoxydibenzoylmethane. These compounds have been demonstrated to not cause harm for tropical corals and their algal symbionts^3^, but detailed information on their effects on other marine organisms is not available yet. The lack of potentially toxic UV filters (benzophenone-3, homosalate, nanoparticles TiO_2_) and the replacement of classical preservatives with sorbic acid in Sunscreen C, can contribute to explain its minimal ecological impact. Previous studies have reported that, being sea urchin larvae very plastic, their anomalies could be also reversible^43, 44^. Therefore, since the alterations observed on the plutei exposed to Sunscreen C was very limited, these larvae had the potential to survive and recover their healthy features.

The altered development of the embryos of *P. lividus* exposed to sunscreens has been reported to be associated with changes in AChE activity. This enzyme is responsible for regulating neurotransmission and other relevant biological processes, including the correct cell migration during gastrulation^20, 45, 46^. In particular, it has been reported that organophosphates pesticides and inorganic nanoparticles act as cholinesterase inhibitors potentially leading to cytoskeletal alterations during the first life stages of sea urchins^20, 21, 34, 35^. We found that all of the three sunscreens tested, determined a significant decrease in cholinesterase activity. The sunscreen ingredients could alter enzymatic activities either by direct inhibition of the AChE enzyme or by binding of its catalytic sites^46^. Our results indicate that AChE activity is highly sensitive to the impact of sunscreens. Indeed, also the product defined “eco-friendly” caused a significant decrease in AChE activity although it determined a low impact on the development of *P. lividus* embryos and larvae.

## Conclusions

The results of the present study provide new insights on the deleterious effects of sunscreen products on marine life revealing the high vulnerability of early developmental stages of sea urchin to such contaminants, and highlighting the potential negative consequences for coastal ecosystem functioning. Our findings show that the three tested sunscreens have a similar efficacy in protecting human fibroblasts from UVA radiation, thus confirming the properties of these sunscreens to screen out the UVA component of sunlight. At the same time, we show that two of the products can have significant impacts on marine organisms. Indeed, commonly used sunscreens, containing chemical filters and preservatives, can severely affect the success of recruitment and survival of the sea urchin through the alteration of biological processes influencing skelotogenesis. Conversely, the sunscreen, in which these compounds were omitted or replaced by other ingredients, had minimal effects. The results presented here underline the importance of developing new eco-friendly products tested on a wide range of marine organisms (including their early life stages, which appear particularly sensitive to the impact of personal care products) for a better preservation of marine life, without renouncing to protect the skin and consequently human health. Since several commercially available sunscreens that display the label “eco-friendly” have not been tested yet on marine organisms, we recommend the development of standardized experimental protocols, based also on the model organism *Paracentrotus*, to assess the impacts of these products on marine life.

## Methods

### Sunscreen Products

We selected three different brands of sunscreens characterized by a high sun protection factor (SPF 40–50+), which is a measure of protection exclusively limited to the UVB wavelengths, and by a different composition in terms of formulation ingredients, UV filters and preservatives, some of which have been demonstrated to impact marine life^3^.

*Sunscreen A* (*SPF 50*+): a product commercially available throughout Europe, containing UV filters in the following order of decreasing concentration: octocrylene, TiO_2_ (nanoparticles), butylmethoxydibenzoylmethane, bis-ethylhexyloxyphenol methoxyphenyl triazine and preservatives (benzyl benzoate).

*Sunscreen B* (*SPF 50*): a popular product commercially available in USA containing UV filters in the following order of decreasing concentration: homosalate, benzophenone-3, octylsalicylate, butylmethoxydibenzoylmethane, octocrylene and preservatives (methylisothiazolinone, methyldibromo glutaronitrile).

*Sunscreen C* (*SPF 40*): a newly patented sunscreen^28^ based on ingredients that have all been tested for protecting marine organisms, including corals and all the marine species depending on them. It contains the following UV filters in order of decreasing concentration: diethylamino hydroxybenzoyl hexyl benzoate, methylene bis-benzotriazolyl tetramethylbutylphenol, ethylhexyl triazone and the preservative sorbic acid.

### Experiments on human dermal fibroblasts (HDF)

#### Cultures of HDF

Primary cultures of human dermal fibroblasts were purchased from Istituto Zooprofilattico Sperimentale, (Brescia, Italy). Human dermal fibroblasts were cultured in Minimum Essential Medium (GIBCO) supplemented with 10% fetal bovine serum (SERA PLUS, PAN biotech GmbH), penicillin (100 U mL^−1^), streptomycin (100 μg mL^−1^) and L-glutamine (2 mM), and maintained in an Heraeus BB15 incubator (Thermo Scientific, Germany) at 37 °C, 5% CO_2_ under humidified atmosphere. Cell culture medium was changed every 2–3 days and fibroblasts were routinely sub-cultured at 80% confluence by trypsinization. For the experiments, cells were seeded at an optimal density of 10 × 10^3^ cells/cm^2^.

#### UVA source

UVA irradiation was provided by a Philips Original Home Solarium sun lamp (model HB 406/A; Philips, Groningen, Holland) equipped with a 400 W ozone-free Philips HPA lamp, UV type 3, delivering a flux of 30.5 mW/cm^2^ between 300 and 400 nm, at a distance of 20 cm from above the samples. The dose of UVA was assessed with a UV Power Pack Radiometer (EIT Inc, Sterling, USA), while the emission spectrum was checked with a StellarNet portable spectroradiometer (Tampa, FL, USA) and is reported elsewhere^47^. Details of the irradiation of sunscreens and analysis of their optical absorption spectra are reported in the Supplementary Information.

#### UVA exposure procedure of human dermal fibroblasts

For irradiation of HDF, the experimental design was similar to the one reported in Brugè *et al*.^48^. Briefly, the cells grown on a 6-well culture dish were washed with phosphate buffered saline (PBS) and covered with a thin layer of PBS prior to exposure. The formulations (2 mg/cm^2^) were spread onto quartz discs (specifically designed for us by Highborn Technology, China) of exactly the same dimensions as the wells of a 6-well cell culture plate and placed on top of the wells prior to irradiation. Sunscreens were therefore not in direct contact with the cells. The cells were then exposed to the UVA source as described above. For the positive control, a quartz disc with no sunscreen was used, while for the negative control the cells were not exposed to UVA.

#### Cell viability and intracellular ROS assay

As indicator of intracellular reactive oxygen species (ROS) formation, the leuco-dye, carboxy-2,7-dichlorofluorescein diacetate (carboxy-H_2_DCFDA) (Invitrogen) was employed as described elsewhere^49^.

The analyses for cell viability and intracellular ROS production were conducted simultaneously on a Guava Easycite flow cytometer (Merck Millipore) using an excitation wavelength of 488 nm. The fluorescence intensity was recorded on an average of 5,000 cells from each sample. Additional details of this procedure are reported in the Supplementary Information.

Specifically, for analyzing the results and to better quantify the differences in intracellular ROS contents, three regions relative to low, mid and high levels of fluorescence corresponding to low, mid and high ROS were defined, based on preliminary experiments on cells exposed to 15 min UVA light. UVA exposure leads to an increase in intracellular ROS formation that can be easily monitored by observing a shift in green fluorescence due to carboxy-DCF: this shift is proportional to ROS formation and was considered as the positive control. Based on the fluorescence distribution between non-exposed and exposed cells, three gates were arbitrarily set, when possible, to define the three regions and the relative percentage of cells in each region was calculated. Counterstaining with Via-count was necessary in order to evaluate intracellular levels of ROS only in viable cells. In fact, exclusion of cells with compromised cell membrane integrity is essential in order to avoid false negatives due to loss of carboxy-H_2_DCFDA from permeable cells.

#### Total RNA extraction and quantitative real-time PCR (qPCR)

After 20 min UVA irradiation, PBS was removed and replaced with medium and cells were incubated for 24 h. Total RNA was then isolated from HDF using the NucleoSpin RNA kit (Macherey-Nagel) according to the manufacturer’s instructions. The RNA purity and concentration were measured on a Nanodrop spectrophotometer. Approximately 400 ng RNA from each sample were then converted to cDNA using the iScript^TM^ cDNA Synthesis Kit (Bio-Rad) according to the manufacturer’s instructions.

qPCR reactions were carried out on a MyiQ Single Color Real-Time PCR Detection System (Bio-Rad) in a 15 µL total reaction volume, using the iQ^TM^ SYBR Green Supermix (Bio-Rad). A minimum of four biological and two technical replicates for each biological replicate were run. The primers were used at a concentration of 400 nM and the sequences for the genes of interest MMP1 and COL1A1, as well as for the reference genes, GAPDH and SDHA previously shown to be the most suitable ones for UVA studies on HDF, are the same as those reported in Brugè *et al*.^50^. The qPCR run was set for a 3 min denaturation step at 95 °C followed by 40 cycles of 15 s denaturation at 95 °C and 30 s of annealing/extension at 60 °C. All PCR efficiencies were between 90 and 110%. The mRNA expression for MMP1 and COL1A1 in UVA treated cells was calculated relative to the expression of these genes in control HDF (non-irradiated), according to the delta-delta Ct method (2^−ΔΔCt^). The results obtained were analysed using the iQ5 Software (Bio-Rad) which automatically gives the normalized fold expression values. The values obtained from at least four independent experiments were then used for statistical analysis using the Mann-Whitney U-test.

### Experiments on *Paracentrotus lividus*

#### Sampling of *P*. *lividus* and spawning induction

Mature specimens of *P*. *lividus* were collected from a coastal area of the Central Adriatic Sea (43°37′11.29″N 13°31′52.9″E, Mediterranean Sea) and immediately transported to the laboratory in refrigerated bags (about 8–10 °C) in wet tissues. In the laboratory, the specimens were maintained in aquaria with filtered seawater (FSW; using 0.22 µm pore size syringe filters, Aisimo^®^) for at least 1 week. The spawning of gametes was obtained as described by Amemiya (1996)^51^ using oral injection of 0.5 M acetylcholine chloride diluted 1:1000 in autoclaved and ultra-filtered seawater (UFSW; using 0.02 µm pore size Anotop syringe filters, Whatman, Springfield Mill, UK, Figure S2). The eggs were collected in sterilized glass containers (n = 6) with 20 mL of FSW, while the sperm was collected dry from the genital pores, divided in aliquots (2 mL) and maintained at 4 °C. Gametes from three different male and three female specimens were mixed. In particular, 130 µL of UFSW containing 3000 eggs (counted under microscope, Zeiss Axioskop, 10× magnification) were mixed with 100 µL of sperms diluted 1:10 with UFSW.

#### Impact of sunscreens on the embryo development of *P. lividus*

The experiments were performed according to the tests validated by ISO and according to Falugi *et al*.^46^. We used 90 sterile tanks (110 mL) containing 100 mL of FSW and 230 µL of mixed gametes at the temperature of 18 °C that is optimal for the synchronous development of urchin eggs^46, 52^. Three sunscreens at different concentrations (10, 20 and 50 µL L^−1^ final concentrations, defined according to the analytical procedures reported in Danovaro *et al*.^3^) were added to three replicated systems (n = 3) for each concentration and compared with untreated systems (without addition of sunscreens, n = 3) used as controls. Sub-samples from each treated (added with sunscreens) and untreated system were collected after the addition of the different concentrations of sunscreens (t_0_ = start of the experiment), after 3 h (corresponding to stage of morula) and 24 h (corresponding to gastrula stage) from the start of the experiment. All sub-samples were fixed with paraformaldehyde (PFA 4%, pH 7.4) and the number of anomalous embryos as well as their morphological characterization were determined within 1 week, on a total of 100 embryos for each system under a light microscope (Zeiss Axioskop, 10× magnification). The use of a sampling design based on independent replicates was needed to avoid pseudo-replication allowing us to perform robust statistical analyses and to increase the information on the natural background variability of the tested model organisms.

#### Impact of sunscreens on larval development of the sea urchin *P. lividus*

The effects of sunscreens on the larval development were assessed in separate experiments from those carried out with embryos. A solution of 15 mL containing *P. lividus* eggs was mixed with 100 µL of diluted sperms (as described above) and incubated at 18 °C in a thermostatic room for 48 h in order to obtain 4-arms larvae of *P. lividus* ^38^. The larvae thus obtained were used as follows. Time-course experiments were performed by using 90 sterile tanks (110 mL) added with 10 mL of UFSW containing *P. lividus* larvae. The different sunscreen products at different concentrations were inoculated as described above for the experiment with embryos. Systems without addition of sunscreens were used as controls. Sub-samples from treated (added with sunscreens) and untreated systems were collected immediately after the addition of sunscreens (t_0_ = start of the experiment) and after 3 and 24 h from the start of the experiment. The sub-samples were fixed with paraformaldehyde (PFA 4%, pH 7.4) and observed under a light microscope (Zeiss Axioskop, 10× magnification) within 1 week in order to determine the number of anomalous larvae over a total of 100 larvae for each sample and their morphological characterization.

#### Morphological analyses of embryos and larvae

The health state of the embryos and larvae were assessed by using a light microscope and classified on the basis of the morphology and synchronicity of embryonic and larval development compared with controls. In detail, embryos were separated into three categories, designated as developed (D), anomalously developed (AD), and non-developed embryos (ND) according to Gambardella *et al*.^38^. D embryos showed normal development, with well-structured archenteron and migratory cells entering the coelom; AD embryos were characterized by defective gastrulae, with typical signs of asymmetrical migration of primary mesenchyme cells and ND embryos showed both an arrested development and gastrulae lacking archenteron and coelom.

Cone-shaped larvae at the *pluteus* stage with four fully developed arms, with complete skeletal rods and with a skeleton of similar size to that of control larvae, were considered as normal larvae according to Carballeira *et al*.^36, 53^. In addition, different types of malformations could be distinguished: crossed, separated tip and fused arms, folded tip and fractured ectoderm, and undeveloped stages. Such malformations were classified according to the degree of larval alteration (level 0: normal development, level 1: incorrect location of skeletal rods, level 2: incomplete or absent skeletal rods, and level 3: development block at the 4-arms *pluteus*), to establish the severity of the sunscreen impact. Therefore, at the end of the experiment (t_24h_), we determined the frequency of anomalies for each degree of larval alteration and calculated the index of sunscreen impact (ISI) as follows:

$${\rm{ISI}}=[0\times  \% \,{\rm{level}}\,0+1\times  \% \,{\rm{level}}\,1+2\times  \% \,{\rm{level}}\,2+3\times  \% \,{\rm{level}}\,3]/100$$

ISI index ranges from 0 (no impact) to 3 (high impact), including also the levels 1 (slight impact) and 2 (moderate impact).

#### Acetylcholinesterase activities

Unfixed samples of *P. lividus* larvae were used to determine acetylcholinesterase activity (AChE, EC, 3.1.1.7) by using the spectrophotometric method^54^. Additional details are reported in the Supplementary Information. AChE activity were expressed as micromoles of substrates hydrolysed per minute per mg of proteins at room temperature.

### Statistical analyses

For the experiments with *P. lividus*, differences in the investigated variables (univariate tests) between controls and treatments, during the experimental time were assessed using permutational analyses of variance (PERMANOVA)^55, 56^. The design included three factors (time, treatment and concentration). When significant differences were encountered (p < 0.05) post-hoc pairwise tests were also carried out. Statistical analyses were performed using the routines included in the PRIMER 6+ software^57^. In the experiments with fibroblasts, significant differences between control and treated samples for cell viability and ROS production were determined using the T-test whereas for gene expression analysis, the Mann-Whitney U test was used.

### Ethics Statement

Farming in aquaria of *P. lividus* was performed in accordance with the best practices developed for the echinoderm species in order to optimize animal health. No specific permissions were required for the locations/activities because *P. lividus* is an invertebrate species, not classified as endangered or protected. All facilities and procedures were compliant with the guidelines of European Union (Directive 609/86).

## Electronic supplementary material

Supplementary methods and figures

## References

[1] Doney SC (2012). Climate change impacts on marine ecosystems. Annual Review of Marine Science.

[2] Halpern BS (2008). A global map of human impact on marine ecosystems. Science.

[3] Danovaro R (2008). Sunscreens cause coral bleaching by promoting viral infections. Environ. Health Perspect..

[4] Calafat AM, Wong LY, Ye X, Reidy JA, Needham LL (2008). Concentrations of the sunscreen agent benzophenone-3 in residents of the United States: National Health and Nutrition Examination Survey 2003-2004. Environ. Health Perspect..

[5] Serra-Roig, M. P. *et al*. Occurrence, fate and risk assessment of personal care products in river–groundwater interface. *Sci. Total Environ*. 1–9 (2016). 10.1016/j.scitotenv.2016.06.006 27320733

[6] Gaw S, Thomas KV, Hutchinson TH (2014). Sources, impacts and trends of pharmaceuticals in the marine and coastal environment. Philos. Trans. R Soc. Lond. B Biol. Sci..

[7] Prichard E, Granek EF (2016). Effects of pharmaceuticals and personal care products on marine organisms: from single-species studies to an ecosystem-based approach. Environ. Sci. Pollut. Res..

[8] Sánchez-Quiles D, Tovar-Sánchez A (2015). Are sunscreens a new environmental risk associated with coastal tourism?. Environ. Int..

[9] Osterwalder U, Sohn M, Herzog B (2014). Global state of sunscreens. Photodermatol. Photoimmunol. Photomed..

[10] McCoshum SM, Schlarb AM, Baum KA (2016). Direct and indirect effects of sunscreen exposure for reef biota. Hydrobiologia.

[11] Diffey BI (2005). Sunscreens and melanoma: the future looks bright. Br J. Dermatol..

[12] Tovar-Sánchez A (2013). Sunscreen products as emerging pollutants to coastal waters. PLoS One.

[13] Díaz-Cruz MS, Barceló D (2009). Chemical analysis and ecotoxicological effects of organic UV-absorbing compounds in aquatic ecosystems. TrAC Trends Anal. Chem..

[14] Langford KH, Thomas KV (2008). Inputs of chemicals from recreational activities into the Norwegian coastal zone. J. Environ. Monit..

[15] Danovaro R, Corinaldesi C (2003). Sunscreen products increase virus production through prophage induction in marine bacterioplankton. Microb. Ecol..

[16] Downs CA (2016). Toxicopathological Effects of the Sunscreen UV Filter, Oxybenzone (Benzophenone-3), on Coral Planulae and Cultured Primary Cells and Its Environmental Contamination in Hawaii and the U.S. Virgin Islands. Arch. Environ. Contam. Toxicol..

[17] Minetto D, Libralato G, Volpi Ghirardini A (2014). Ecotoxicity of engineered TiO_2_ nanoparticles to saltwater organisms: An overview. Environ. Int..

[18] Sánchez-Quiles D, Tovar-Sánchez A (2014). Sunscreens as a source of hydrogen peroxide production in coastal waters. Environ. Sci. Technol..

[19] Narayanan DL, Saladi RN, Fox JL (2010). Review: Ultraviolet radiation and skin cancer. Int. J. Dermatol..

[20] Gambardella C (2015). Multidisciplinary screening of toxicity induced by silica nanoparticles during sea urchin development. Chemosphere.

[21] Mesaric T (2015). Sperm exposure to carbon-based nanomaterials causes abnormalities in early development of purple sea urchin (Paracentrotus lividus). Aquat. Toxicol..

[22] Duran, I., Bikfalvi, A. & Llach, J. New facets of quality. A multiple case study of Green cosmetic manufacturers. *European Accounting and Management Review* 1(1) (2014).

[23] Fent K, Kunz PY, Zenker A, Rapp M (2010). A tentative environmental risk assessment of the UV-filters 3-(4-methylbenzylidene-camphor), 2-ethyl-hexyl-4-trimethoxycinnamate, benzophenone-3, benzophenone-4 and 3-benzylidene camphor. Mar. Environ. Res..

[24] Kim S, Choi K (2014). Occurrences, toxicities, and ecological risks of benzophenone-3, a common component of organic sunscreen products: a mini-review. Environ. Int..

[25] Maipas S, Nicolopoulou-Stamati P (2015). Sun lotion chemicals as endocrine disruptors. Hormones.

[26] Paredes E, Perez S, Rodil R, Quintana JB, Beiras R (2014). Ecotoxicological evaluation of four UV filters using marine organisms from different trophic levels Isochrysis galbana, Mytilus galloprovincialis, Paracentrotus lividus, and Siriella armata. Chemosphere.

[27] Manzo S, Miglietta ML, Rametta G, Buono S, Di Francia G (2013). Embryotoxicity and spermiotoxicity of nanosized ZnO for Mediterranean sea urchin Paracentrotus lividus. J. Hazard. Mater..

[28] Danovaro, R., Damiani, E. & Corinaldesi, C. Sunscreen compositions: www.google.ch/patents/ WO 2013014584 A2 (2014).

[29] Dondi D, Albini A, Serpone N (2006). Interactions between different solar UVB/UVA filters contained in commercial sunscreens and consequent loss of UV protection. Photochem. Photobiol. Sci..

[30] Gonzalez H (2007). Photostability of commercial sunscreens upon sun exposure and irradiation by ultraviolet lamps. BMC dermatol..

[31] Wondrak GT, Jacobson MK, Jacobson EL (2006). Endogenous UVA-photosensitizers: mediators of skin photodamage and novel targets for skin photoprotection. Photochem. Photobiol. Sci..

[32] Burnett ME, Wang SQ (2011). Current sunscreen controversies: A critical review. Photodermatol. Photoimmunol. Photomed..

[33] Buechner N (2008). Changes of MMP-1 and collagen typeI-alpha 1 by UVA, UVB and IRA are differentially regulated by Trx-1. Exp Gerontol.

[34] Pesando D (2003). Biological targets of neurotoxic pesticides analysed by alteration of developmental events in the Mediterranean sea urchin. Paracentrotus lividus. Mar. Environ. Res..

[35] Aluigi MG, Falugi C, Mugno MG, Privitera D, Chiantore M (2010). Dose-dependent effects of chlorpyriphos, an organophosphate pesticide, on metamorphosis of the sea urchin. Paracentrotus lividus. Ecotoxicology.

[36] Carballeira C, Ramos-Gomez J, Martin-Diaz L, DelValls TA (2012). Identification of specific malformations of sea urchin larvae for toxicity assessment: Application to marine pisciculture effluents. Mar. Environ. Res..

[37] Peterson RE, McClay DR (2003). Primary mesenchyme cell patterning during the early stages following ingression. Dev. Biol..

[38] Gambardella C (2013). Developmental abnormalities and changes in cholinesterase activity in sea urchin embryos and larvae from sperm exposed to engineered nanoparticles. Aquat. Toxicol..

[39] Guillou M, Quiniou F, Huart B, Pagano G (2000). Comparison of embryonic development and metal contamination in several populations of the sea urchin *Sphaerechinus granularis* (Lamarck) exposed to anthropogenic pollution. Arch. Environ. Contam. Toxicol..

[40] Krause M (2012). Sunscreens: Are they beneficial for health? An overview of endocrine disrupting properties of UV-filters. Int. J. Androl..

[41] Schlumpf M (2004). Endocrine activity and developmental toxicity of cosmetic UV filters–an update. Toxicology.

[42] Kunz PY, Fent K (2006). Multiple hormonal activities of UV filters and comparison of *in vivo* and *in vitro* estrogenic activity of ethyl-4-aminobenzoate in fish. Aquat. Toxicol..

[43] Ruocco N, Costantini M, Santella L (2016). New insights into negative effects of lithium on sea urchin *Paracentrotus lividus* embryos. Sci. Rep..

[44] Castellano I (2016). Shedding light on ovothiol byosinthesis in marine metazoans. Sci. Rep..

[45] Drews U (1975). Cholinesterase in embryonic development. Prog. Histochem. Cytochem..

[46] Falugi C, Lammerding-Koppel M, Aluigi MG (2008). Sea urchin development: An alternative model for mechanistic understanding of neurodevelopment and neurotoxicity. *Birth Defects Res*. Part C - Embryo Today Rev..

[47] Venditti E (2008). *In vitro* photostability and photoprotection studies of a novel ‘multi-active’ UV-absorber. Free Radic. Biol. Med.

[48] Brugè F, Tiano L, Astolfi P, Emanuelli M, Damiani E (2014). Prevention of UVA-induced oxidative damage in human dermal fibroblasts by new UV filters, assessed using a novel *in vitro* experimental system. PloS one.

[49] Brugè F (2015). A comparative study on the possible cytotoxic effects of different nanostructured lipid carrier (NLC) compositions in human dermal fibroblasts. Int. J. Pharm..

[50] Brugè F, Venditti E, Tiano L, Littarru GP, Damiani E (2011). Reference gene validation for qPCR on normoxia- and hypoxia-cultured human dermal fibroblasts exposed to UVA: is beta-actin a reliable normalizer for photoaging studies?. J. Biotechnol..

[51] Amemiya S (1996). Complete regulation of development throughout metamorphosis of sea urchin embryos devoid of macromeres. Dev. Growth Differ..

[52] Privitera D, Noli M, Falugi C, Chiantore M (2011). Benthic assemblages and temperature effects on *Paracentrotus lividus* and *Arbacia lixula* larvae and settlement. J. Exp. Mar. Biol. Ecol..

[53] Carballeira C, De Orte MR, VianaI IG, Carballeira A (2011). Implementation of a minimal biological test set for assessment of ecotoxic effect of effluents from land-based fish farms. Ecotoxicology and Environmental Safety.

[54] Ellman GL, Courtney KO, Andres V, Featherstone RM (1961). A new and rapid colorimetric determination of acetylcholinesterase activity. Biochem. Pharmaco.l.

[55] Anderson, M. J. Permutational multivariate analysis of variance. Department of Statistics, University of Auckland, Auckland (2005).

[56] McArdle BH, Anderson MJ (2001). Fitting multivariate models to community data: a comment on distance-based redundancy analysis. Ecology.

[57] Clarke, K. R. & Gorley, R. N. PRIMER v6: User Manual/Tutorial. PRIMER-E, Plymouth (2006).

