# Supplementary material for: Sunscreen products impair the early developmental stages of the sea urchin Paracentrotus lividus
Source: Sci Rep. 2017 Aug 10;7:7815. doi: 10.1038/s41598-017-08013-x (PMC5552690; doi:10.1038/s41598-017-08013-x)
Supplement: Supplementary file 1 — Supplementary methods and figures [file 41598_2017_8013_MOESM1_ESM.pdf]

Supplementary information

SREP-17-10129

Sunscreen products impair the early developmental stages of the sea urchin *Paracentrotus lividus*

Cinzia Corinaldesi[1*], Elisabetta Damiani[2*], Francesca Marcellini[2,3], Carla Falugi[2], Luca Tiano[2], Francesca Brugè[4], Roberto Danovaro[2,5]

[1] Department of Sciences and Engineering of Materials, Environment and Urbanistics, Polytechnic University of Marche, Via Brecce Bianche, Ancona, Italy

[2] Department of Life and Environmental Sciences, Polytechnic University of Marche, Via Brecce Bianche, Ancona, Italy

[3] Ecoreach Ltd., Corso Stamira 61, 60121, Ancona, Italy

[4] Department of Clinical, Specialistic and Odontostomatological Sciences, Polytechnic University of Marche, Via Brecce Bianche, Ancona, Italy

[5] Stazione Zoologica Anton Dohrn, Naples, Italy

* Contributed equally to this work

Supplementary methods

UVA exposure procedure of sunscreens and optical absorption spectra

For irradiation of sunscreens, 50±2 mg of sunscreen, as recommended by the COLIPA (2006) sun protection factor test method[1], were spread onto 25 cm^2 glass plates with a gloved finger and left to dry for 30 min in the dark. The plates were placed on a brass block set on ice and exposed to UVA for 15 min, i.e. 275 kJm^{-2} (equivalent to *circa* 90 min of sunshine at the French Riviera (Nice) in summer at noon[2]. The unexposed samples were kept in the dark for the same amount of time as the exposed ones. After irradiation of sunscreens, the glass plates were placed in beakers and immersed in 10 mL ethyl acetate for 30 min with manual shaking every 10 min for maximum extraction of the UV-filters. From the organic solution, 0.05 mL were added to 2.45 mL ethyl acetate in a quartz cuvette and its absorption spectrum was measured against a blank containing ethyl acetate, on a Shimadzu UV-2401PC spectrophotometer against a blank containing ethyl acetate.

Cell viability and intracellular ROS assay

The leuco-dye, carboxy-2,7-dichlorofluorescein diacetate (carboxy-H_2DCFDA) (Invitrogen) was used as indicator of intracellular reactive oxygen species (ROS) formation as described elsewhere[3]. Briefly, after irradiation PBS was removed from the cell culture wells and replaced with either cell culture medium followed by incubation of the cells for 24 h (for assessment 24 h post-irradiation), or fresh PBS. Subsequently, 10 μM carboxy-H_2DCFDA solution in PBS was added to each sample and incubated for 30 min in the dark at 37°C for immediate analysis. After removing the dye, the cells were washed with PBS, trypsinized, harvested, centrifuged and the cell pellet was resuspended in 100 μL PBS. An aliquot of 30 μL from each sample was then added to 270 μL of a Guava Via-count solution (Merck Millipore)

diluted 1/5 with PBS. This is a fluorescent stain formulation, which provides sensitive and accurate detection of viable, apoptotic and dead cells in flow cytometry. The analyses for cell viability and intracellular ROS production were conducted simultaneously on a Guava Easycite flow cytometer (Merck Millipore) using an excitation wavelength of 488 nm. Emissions were recorded using the green channel for carboxy-DCF (the oxidized form of carboxy-H_2DCFDA) and the red and yellow channels for the Via-count dye, using the following gain settings: FSC 20.7; SSC 9.93; GRN 3.51; YEL 15.3; RED 11.8 and a threshold of 1000 on FSC.

Acetylcholinesterase activities

Unfixed samples of *P. lividus* larvae were used to determine acetylcholinesterase activity (AChE, EC, 3.1.1.7) by using the spectrophotometric method[4]. Such an activity was measured in *P. lividus* larvae (n=200) collected from treated and untreated systems (n=3) immediately after sunscreen addition (50 µL L^{-1}) and after 3 h and 24 h incubations. Larvae were frozen after each time point overnight. The frozen samples were then thawed, homogenized with a minipotter (B. Braun Melsunger), passed through a syringe with a thin needle, (Ultrafin 29G, 12.7 mm length), in the presence of 1% Triton X100, sonicated for 25 min (Branson, 3510) and centrifuged for 3 min at 8000 rpm. The supernatants were used to determine AChE at $\lambda = 412$ nm. The kinetic of AChE activity was obtained by measuring the velocity of substrate cleavage for 3 min compared with the linear equation of a standard curve that had been previously obtained by supplying known amounts of ChEs[5]. The protein content in the supernatants of untreated and treated samples was measured using the method described by Lowry et al. (1951)[6],

subsequently modified by Hartree (1972)[7]. The AChE units were obtained by the ratio between the micromoles of substrates hydrolyzed/min/mg proteins at room temperature.

Supplementary figures

Figure S1. UV absorption spectra of sunscreens, before and after UVA exposure (275 kJ/m^2) followed by extraction with ethyl acetate. A=European sunscreen, SPF 50+; B=USA sunscreen, SPF 50; C=Eco-friendly sunscreen, SPF 40, D=comparison of all three spectra.

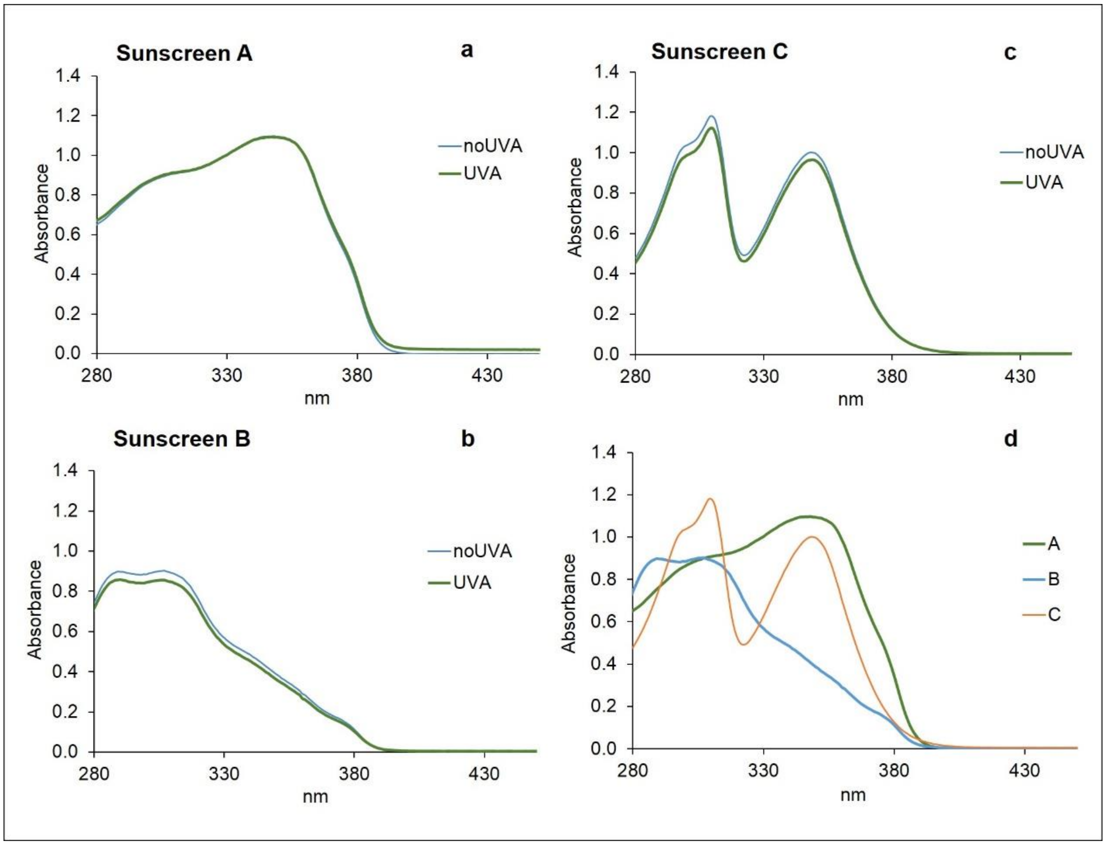

References

1. COLIPA Guidelines: International Sun Protection Factor (SPF) test method 46 p. (2006).
2. Séite, S. *et al*. Mexoryl® SX: a broad absorption UVA filter protects human skin from the effects of repeated suberythemal doses of UVA. *J. Photochem. Photobiol. B* **44**, 69-76 (1998)
3. Brugè, F., Damiani, E., Marcheggiani, F., Offerta, A., Puglia, C. & Tiano, L. A comparative study on the possible cytotoxic effects of different nanostructured lipid carrier (NLC) compositions in human dermal fibroblasts. *Int. J. Pharm.* **495**,879-885 (2015).
4. Ellman, G. L., Courtney, K. O., Andres, V. & Featherstone, R. M. A new and rapid colorimetric determination of acetylcholinesterase activity. *Biochem. Pharmacol.* **7**, 88- 95 (1961).
5. Gambardella, C. *et al.* Developmental abnormalities and changes in cholinesterase activity in sea urchin embryos and larvae from sperm exposed to engineered nanoparticles. *Aquat. Toxicol.* **130–131,** 77–85 (2013).
6. Lowry, O. H., Rosebrough, N. J., Farr, L. & Randall, R. J. Protein measurement with the folin phenol reagent. *J. Biol. Chem.* **193**, 265-275 (1951).
7. Hartree, E. F. Determination of proteins: a modification of the Lowry method that give a linear photometric response. *Anal. Biochem.* **48**, 422–7 (1972).
